# ProMEX: a mass spectral reference database for proteins and protein phosphorylation sites

**DOI:** 10.1186/1471-2105-8-216

**Published:** 2007-06-23

**Authors:** Jan Hummel, Michaela Niemann, Stefanie Wienkoop, Waltraud Schulze, Dirk Steinhauser, Joachim Selbig, Dirk Walther, Wolfram Weckwerth

**Affiliations:** 1 Max Planck Institute of Molecular Plant Physiology, Am Mühlenberg 1, 14424 Potsdam, Germany; 2 University of Potsdam, Institute of Biochemistry and Biology, c/o MPI-MP, Am Mühlenberg 1, 14424 Potsdam, Germany

## Abstract

**Background:**

In the last decade, techniques were established for the large scale genome-wide analysis of proteins, RNA, and metabolites, and database solutions have been developed to manage the generated data sets. The Golm Metabolome Database for metabolite data (GMD) represents one such effort to make these data broadly available and to interconnect the different molecular levels of a biological system [1]. As data interpretation in the light of already existing data becomes increasingly important, these initiatives are an essential part of current and future systems biology.

**Results:**

A mass spectral library consisting of experimentally derived tryptic peptide product ion spectra was generated based on liquid chromatography coupled to ion trap mass spectrometry (LC-IT-MS). Protein samples derived from *Arabidopsis thaliana*, *Chlamydomonas reinhardii*, *Medicago truncatula*, and *Sinorhizobium meliloti *were analysed. With currently 4,557 manually validated spectra associated with 4,226 unique peptides from 1,367 proteins, the database serves as a continuously growing reference data set and can be used for protein identification and quantification in uncharacterized biological samples. For peptide identification, several algorithms were implemented based on a recently published study for peptide mass fingerprinting [2] and tested for false positive and negative rates. An algorithm which considers intensity distribution for match correlation scores was found to yield best results. For proof of concept, an LC-IT-MS analysis of a tryptic leaf protein digest was converted to mzData format and searched against the mass spectral library. The utility of the mass spectral library was also tested for the identification of phosphorylated tryptic peptides. We included *in vivo *phosphorylation sites of *Arabidopsis thaliana *proteins and the identification performance was found to be improved compared to genome-based search algorithms. Protein identification by ProMEX is linked to other levels of biological organization such as metabolite, pathway, and transcript data. The database is further connected to annotation and classification services via BioMoby.

**Conclusion:**

The ProMEX protein/peptide database represents a mass spectral reference library with the capability of matching unknown samples for protein identification. The database allows text searches based on metadata such as experimental information of the samples, mass spectrometric instrument parameters or unique protein identifier like AGI codes. ProMEX integrates proteomics data with other levels of molecular organization including metabolite, pathway, and transcript information and may thus become a useful resource for plant systems biology studies. The ProMEX mass spectral library is available at .

## Background

Large-scale protein analysis is closely linked to mass spectrometric techniques. Peptide fragmentation in an ion trap mass spectrometer (IT-MS) is one of the most used approaches for protein identification in complex samples [3]. One such technique, referred to as "shotgun proteomics", can be exploited for rapid screening and – in combination with further fractionation – comprehensive qualitative protein identification in complex samples [4,5]. Proteins are extracted from tissues and analysed after separation on gel electrophoresis or directly via tryptic digestion. Thousands or tens of thousands of peptide mass spectra are generated in a typical analysis. For high throughput identification, these mass spectra can be matched against theoretical amino acid sequences derived from the annotated or predicted proteome of an organism. However, in many of the available algorithms, only the appearance of specific mass fragments in a CID-MS, but not their intensity distribution is considered specifically, because it can not easily be predicted from the amino acid sequence of peptides alone. Therefore, the approach of establishing a mass spectral reference library as used routinely in metabolic and metabolite profiling by gas chromatography coupled to mass spectrometry appears promising [6-8]. We investigate here the utility of a mass spectral reference library by implementing a database consisting of 4,557 manually validated tryptic peptide product ion spectra of divers plant proteins generated by LC-IT-MS and mass fragment intensity correlation search for protein identification.

## Construction and content

### Creation of the mass spectral library

A library of 4,557 manually validated peptide/phosphopeptide product ion spectra from approximately 330,000 collision-induced dissociation (CID) fragment spectra analysed in various LC-MS/MS analyses from recent studies was generated [4,5,9-12]. These CID spectra were annotated with Sequest and Mascot using the filter and search criteria as described in these studies [4,5,9-12]. In addition to the filtering, the resulting spectra were further validated manually using the SILVER web application [13].

### ProMEX database system

ProMEX was designed as a web-based service in a Linux/Apache environment using Perl-CGI for HTML page generation. A Microsoft SQL Server 2005^® ^was utilised as the relational database backend for storing the library spectra as well as the query spectra uploaded by the user. Algorithms for comparing spectra to identify matching hits were implemented using the Common Language Runtime (CLR) .Net framework using the *C# *programming language. Library mass spectra as well as user-uploaded query spectra are saved using a User-defined Data Type (UDT) in the database permitting us to calculate the score values directly within the database by using standard T-SQL to access the spectra. Using indices to select matching library spectra according to a given precursor ion mass and a charge state of a user-submitted product ion spectra and taking advantage of the server's memory management system, we achieved high performance and efficient memory usage.

**Figure 1:**
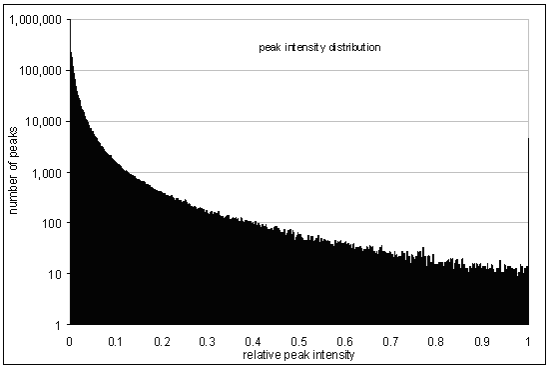
Peak intensity distribution from product ion spectra. 70% of all fragment peaks have a relative intensity less or equal 0.02. The peak on the right hand side originates from the prior scaling of all spectra to a maximum peak intensity of 1.

**Figure 2:**
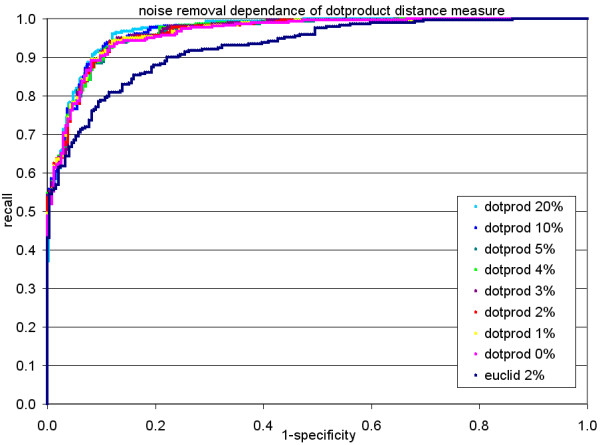
Assessment of the effect of noise removal on the identification performance illustrated by the receiver operating characteristic (ROC). The identification rate is robust against different levels of peak noise filters up to 10%. We decided to use a conservative noise removal level of 2%. Recall (true positives) reflects the number of correctly identified peptides based on SEQUEST search, whereas 1 specificity corresponds to all ProMEX peptide identifications which did not match to the SEQUEST identifications. As a comparison, results from using the Euclidean distance are also included which proved to be inferior to the dot product.

Because of the server's limitation of the UDT size to 8,000 Bytes, it was necessary to limit the product ion spectra to maximally 1,000 fragment peaks each (1,000 peaks with 4 Byte *m/z *and 4 Byte *intensity *each). Upon evaluating the intensity distribution (Figure 1), we assessed the dependence of the identification process on the level of noise removal; i.e. peaks with very low intensity. Applying the receiver operating characteristic (ROC) analysis, peptide identification accuracy was observed to be robust with regard to noise filtering and a level of 2% relative peak intensity was chosen as a conservative threshold for noise removal (Figure 2).

As described in [2], the performance of peak-list comparison is influenced by several factors, most importantly by the choice of the distance measurement itself. Here, we found a modified dot product [7] distance measure

D(x,y)=1−∑i∈A,k∈Bw((mz)ix,(mz)ky)I^ixI^ky
 MathType@MTEF@5@5@+=feaafiart1ev1aaatCvAUfKttLearuWrP9MDH5MBPbIqV92AaeXatLxBI9gBaebbnrfifHhDYfgasaacH8akY=wiFfYdH8Gipec8Eeeu0xXdbba9frFj0=OqFfea0dXdd9vqai=hGuQ8kuc9pgc9s8qqaq=dirpe0xb9q8qiLsFr0=vr0=vr0dc8meaabaqaciaacaGaaeqabaqabeGadaaakeaacqWGebarcqGGOaakcqWG4baEcqGGSaalcqWG5bqEcqGGPaqkcqGH9aqpcqaIXaqmcqGHsisldaaeqbqaaiabdEha3naabmaabaWaaeWaaeaadaWccaqaaiabd2gaTbqaaiabdQha6baaaiaawIcacaGLPaaadaqhaaWcbaGaemyAaKgabaGaemiEaGhaaOGaeiilaWYaaeWaaeaadaWccaqaaiabd2gaTbqaaiabdQha6baaaiaawIcacaGLPaaadaqhaaWcbaGaem4AaSgabaGaemyEaKhaaaGccaGLOaGaayzkaaGafmysaKKbaKaadaqhaaWcbaGaemyAaKgabaGaemiEaGhaaOGafmysaKKbaKaadaqhaaWcbaGaem4AaSgabaGaemyEaKhaaaqaaiabdMgaPjabgIGiolabdgeabjabcYcaSiabdUgaRjabgIGiolabdkeacbqab0GaeyyeIuoaaaa@5C52@

to perform best on the mass spectra used in this study where *x *and *y *denote the two spectra to be compared, (mz)ix
 MathType@MTEF@5@5@+=feaafiart1ev1aaatCvAUfKttLearuWrP9MDH5MBPbIqV92AaeXatLxBI9gBaebbnrfifHhDYfgasaacH8akY=wiFfYdH8Gipec8Eeeu0xXdbba9frFj0=OqFfea0dXdd9vqai=hGuQ8kuc9pgc9s8qqaq=dirpe0xb9q8qiLsFr0=vr0=vr0dc8meaabaqaciaacaGaaeqabaqabeGadaaakeaadaqadaqaamaaliaabaGaemyBa0gabaGaemOEaOhaaaGaayjkaiaawMcaamaaDaaaleaacqWGPbqAaeaacqWG4baEaaaaaa@3428@ are the mass-charge ratios and I^ix
 MathType@MTEF@5@5@+=feaafiart1ev1aaatCvAUfKttLearuWrP9MDH5MBPbIqV92AaeXatLxBI9gBaebbnrfifHhDYfgasaacH8akY=wiFfYdH8Gipec8Eeeu0xXdbba9frFj0=OqFfea0dXdd9vqai=hGuQ8kuc9pgc9s8qqaq=dirpe0xb9q8qiLsFr0=vr0=vr0dc8meaabaqaciaacaGaaeqabaqabeGadaaakeaacuWGjbqsgaqcamaaDaaaleaacqWGPbqAaeaacqWG4baEaaaaaa@30D8@ are the normalised intensity values of the *i-*th peak in the spectrum *x*, likewise for spectrum *y *and index *k*. *A *and *B *are the total number of peaks in the spectra *x *and *y*, respectively. The term

w((mz)ix,(mz)ky)={1−|(mz)ix−(mz)ky|a;|(mz)ix−(mz)ky|≤a0;|(mz)ix−(mz)ky|>a
 MathType@MTEF@5@5@+=feaafiart1ev1aaatCvAUfKttLearuWrP9MDH5MBPbIqV92AaeXatLxBI9gBaebbnrfifHhDYfgasaacH8akY=wiFfYdH8Gipec8Eeeu0xXdbba9frFj0=OqFfea0dXdd9vqai=hGuQ8kuc9pgc9s8qqaq=dirpe0xb9q8qiLsFr0=vr0=vr0dc8meaabaqaciaacaGaaeqabaqabeGadaaakeaacqWG3bWDdaqadaqaamaabmaabaWaaSGaaeaacqWGTbqBaeaacqWG6bGEaaaacaGLOaGaayzkaaWaa0baaSqaaiabdMgaPbqaaiabdIha4baakiabcYcaSmaabmaabaWaaSGaaeaacqWGTbqBaeaacqWG6bGEaaaacaGLOaGaayzkaaWaa0baaSqaaiabdUgaRbqaaiabdMha5baaaOGaayjkaiaawMcaaiabg2da9maaceaabaqbaeqabiGaaaqaaiabigdaXiabgkHiTmaalaaabaWaaqWaaeaadaqadaqaamaaliaabaGaemyBa0gabaGaemOEaOhaaaGaayjkaiaawMcaamaaDaaaleaacqWGPbqAaeaacqWG4baEaaGccqGHsisldaqadaqaamaaliaabaGaemyBa0gabaGaemOEaOhaaaGaayjkaiaawMcaamaaDaaaleaacqWGRbWAaeaacqWG5bqEaaaakiaawEa7caGLiWoaaeaacqWGHbqyaaaabaGaei4oaSZaaqWaaeaadaqadaqaamaaliaabaGaemyBa0gabaGaemOEaOhaaaGaayjkaiaawMcaamaaDaaaleaacqWGPbqAaeaacqWG4baEaaGccqGHsisldaqadaqaamaaliaabaGaemyBa0gabaGaemOEaOhaaaGaayjkaiaawMcaamaaDaaaleaacqWGRbWAaeaacqWG5bqEaaaakiaawEa7caGLiWoacqGHKjYOcqWGHbqyaeaacqaIWaamaeaacqGG7aWodaabdaqaamaabmaabaWaaSGaaeaacqWGTbqBaeaacqWG6bGEaaaacaGLOaGaayzkaaWaa0baaSqaaiabdMgaPbqaaiabdIha4baakiabgkHiTmaabmaabaWaaSGaaeaacqWGTbqBaeaacqWG6bGEaaaacaGLOaGaayzkaaWaa0baaSqaaiabdUgaRbqaaiabdMha5baaaOGaay5bSlaawIa7aiabg6da+iabdggaHbaaaiaawUhaaaaa@86A6@

is a windowed, linear weighting factor accounting for the match accuracy with regard to mass-charge ratio difference of fragment peaks *x*_*i *_and *y*_*k *_where *a *corresponds to the *fragment ion mass tolerance *specified by the user. The same strategy was applied in a recent mass spectral match algorithm [2]. Because all peaks are normalised to

I^=I∑j=1lIj2
 MathType@MTEF@5@5@+=feaafiart1ev1aaatCvAUfKttLearuWrP9MDH5MBPbIqV92AaeXatLxBI9gBaebbnrfifHhDYfgasaacH8akY=wiFfYdH8Gipec8Eeeu0xXdbba9frFj0=OqFfea0dXdd9vqai=hGuQ8kuc9pgc9s8qqaq=dirpe0xb9q8qiLsFr0=vr0=vr0dc8meaabaqaciaacaGaaeqabaqabeGadaaakeaacuWGjbqsgaqcaiabg2da9maalaaabaGaemysaKeabaWaaOaaaeaadaaeWbqaaiabdMeajnaaDaaaleaacqWGQbGAaeaacqaIYaGmaaaabaGaemOAaOMaeyypa0JaeGymaedabaGaemiBaWganiabggHiLdaaleqaaaaaaaa@3AA5@

where *I *refers to the raw intensities of a given spectrum with *l *peaks including only those peaks above the noise threshold discussed above, *D *is always within the range of (0, 1) with values closer to zero indicating better matches between two spectra. The dot product distance is calculated looping through both MS/MS spectra *x *and *y *under comparison sorted in ascending order according to their *m/z *ratios and using a reference pointing to the next fragment peak for a potential fragment match for each peak list. In case the mass difference between those referenced peaks is less than or equal to the fragment ion mass tolerance *a*, both referenced peaks are used in Eq. 1 with their respective intensities. Otherwise a zero-intensity peak is "matched" to that referenced peak. In the first case, both peak reference pointers are incremented simultaneously, whereas only the index pointing to the peak with the smaller *m/z *ratio is incremented in the latter case. We iterate that loop until all peaks are checked off.

**Figure 3:**
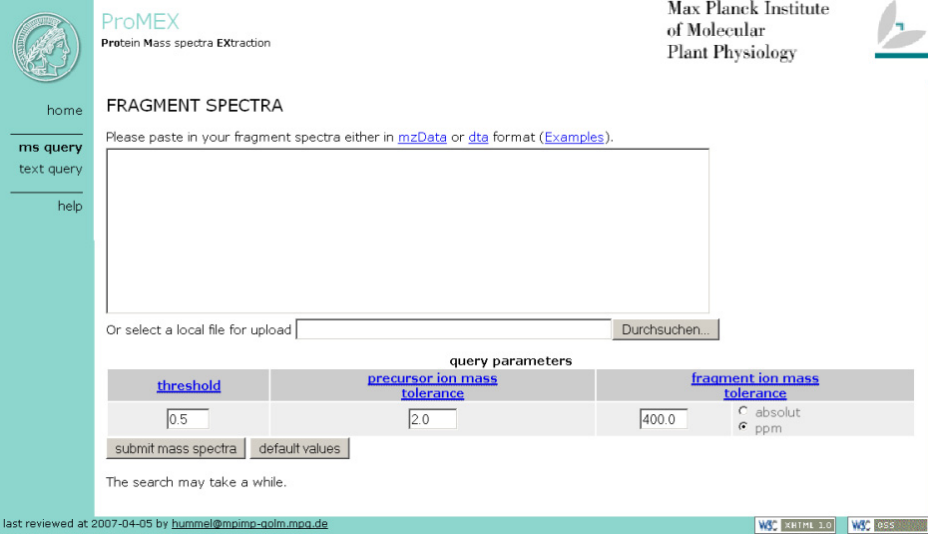
Screenshot of the ProMEX user interface. Input can be inserted into the textbox or uploaded as file using the file selection dialog.

To balance flexible process parameterisation and performance of mass spectra identification, we limited the user interface to a subset of parameters (Figure 3):

• *Precursor ion mass tolerance *defines a window for considering two distinct MS/MS spectra coming from the same parent ion;

• *Fragment ion mass tolerance *defines a window for considering two ion peaks from different peak lists as identical;

• *Threshold *defines a threshold value to ignore mass spectra hits above this score value; i.e. spectra are not considered as matching.

**Figure 4:**
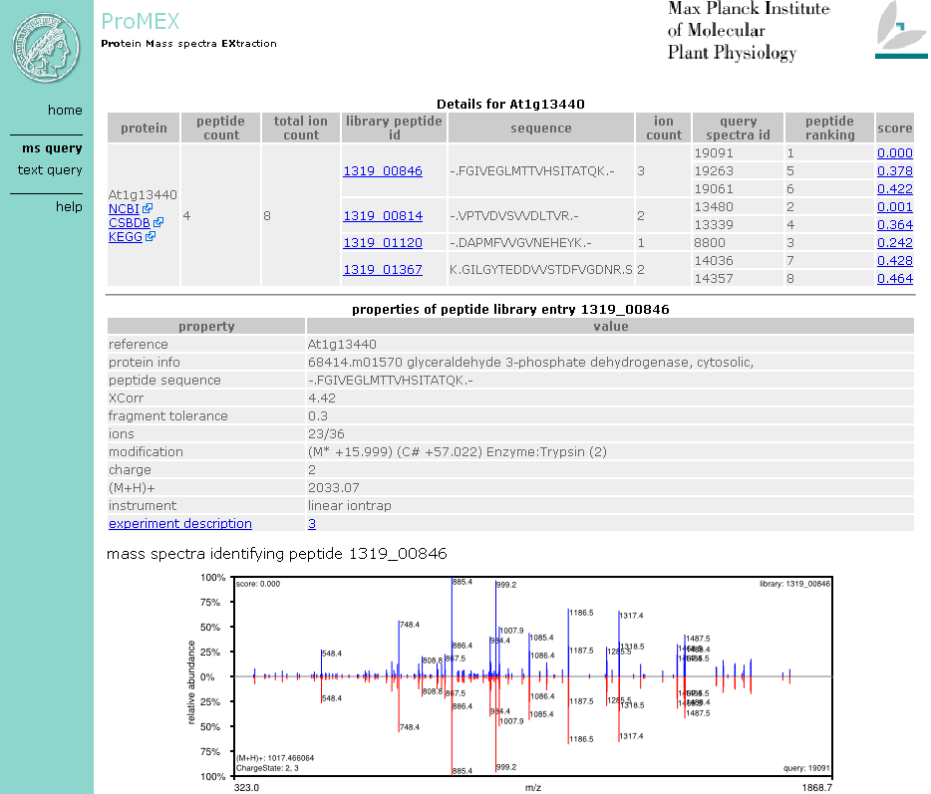
Screenshot of the result page showing four identified peptides associated with the candidate hit protein *At1g13440 *which was tagged in the sample by 8 submitted fragment spectra. For visual inspection of the spectra match, a north-south-plot of the library versus query spectra is shown below.

ProMEX is able to handle uploads of merged MS/MS text files (dta or mgf) [14] or mzData  standard . User-uploaded data are verified and parsed using Perl, and the spectra submitted as queries to the database. Spectra contained in the library are sorted according to their matching score ignoring hits above a given score threshold value. The hit list of identified proteins is further augmented by information contained in the database including peptide annotations, information regarding the biological experiment, instrument settings as well as links to online resources of the National Center for Biotechnology Information (NCBI), the Kyoto Encyclopedia of Genes and Genomes (KEGG), and the Comprehensive Systems-Biology Database (CSB.DB) (Figure 4).

Considering widespread use of the BioMOBY  environment in web-based bioinformatics and as a common framework for biological resources, we implemented a service *getTrypticPeptideSequenceByAGI *that returns a list of experimentally measured tryptic peptides for *Arabidopsis *genes identified by their *Arabidopsis *Genome Initiative (AGI) code.

To obtain maximal performance and limit temporary disk space usage, all uploaded spectra, results, and session information will be deleted within 24 hours after transmittal. Using current standard hardware equipment, a typical ProMEX analysis of a 196 MB merged dta file with >30,000 MS/MS spectra is completed within approximately 13 minutes.

## Utility and Discussion

### Mass spectral library and experimental metainformation

A library containing 4,557 measured peptide MS/MS spectra from 1,367 distinct proteins represented by 4,226 peptides including the corresponding experimental information was created. In case of replicated fragment spectra contained in the database – a small fraction of all spectra, only the best matching spectrum is returned. In addition to existing algorithms which use mass spectral library searches such as GPM X!Hunter  or SpectraST [6], we attempted to link the identification process to experimental metainformation and quantification. The latter is facilitated by the possibility to upload complete LC-MS runs in mzData format and to search them against the database. The result of peptide/protein identifications is accompanied by a semiquantitative measure called spectral count giving the possibility to estimate the relative abundance of proteins in the sample [4,15] (see also below). Furthermore, we optimised the database search to allow analyses of large file sizes (up to 200 Mbyte and more, see below) which to our knowledge is not yet implemented in other databases. The peptide spectra presently stored in the database were mainly derived from *Arabidopsis thaliana*, *Medicago truncatula*, *Chlamydomonas reinhardii *and *Sinorhizobium meliloti*, acquired in 18 experiments through fragmentation with LCQ, LTQ or Orbitrap mass spectrometers [5,9,10,12].

### ProMEX mass spectral library search of unknown samples

For the analysis of protein identification efficiency of ProMEX, LC/MS analyses were processed by both ProMEX and SEQUEST . The disadvantage of ProMEX may lie in the use of an inherently incomplete dataset compared to a genomic database. On the other hand, a mass spectral library consisting of experimentally observed and validated spectra as implemented in ProMEX can be expected to provide clear advantages over genome-based prediction of mass spectra [6].

**Figure 5:**
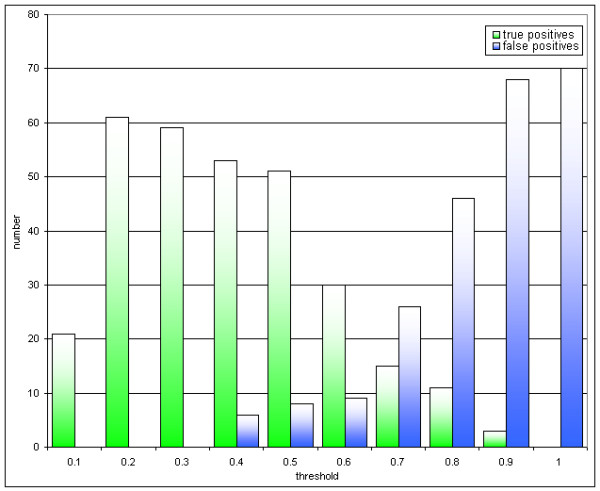
Comparison of peptide identification rate as a function of match score. True positive identifications are all matches found by SEQUEST and ProMEX. False positives are defined as residual peptide identifications by ProMEX which did not match to the SEQUEST hits. The default threshold value for ProMEX was set to 0.5 according to this diagram.

To set a suitable default cutoff threshold for the search algorithm implemented in ProMEX, two different distance measures – the dot product and Euclidean distance – were compared by searching mzData files of LC-MS analyses against the ProMEX mass spectral library. The results were compared to the SEQUEST result file ("true positives"). Figure 2 illustrates the gain of correct predictions by the dot product in comparison to the Euclidean distance. Because of the better performance, the dot product distance was chosen and the default threshold value was set to 0.5. In Figure 5, the resulting peptide identification output was examined for the number of false hits against true positive peptide hits (list of peptides identified by SEQUEST) as a function of threshold score for the dot product distance. Using the default threshold, the false peptide detection rate was found to be less than 1%, while the number of false negatives reached a level of 25% in our dataset.

For 86 proteins, the list of protein identifications was identical in both systems. From subsequent searches, it became clear that the specificity for peptide identification increases with ProMEX because fragment intensity distributions in product ion spectra are taken into account as an additional parameter.

Very importantly, spectra which are not assigned by SEQUEST or MASCOT can easily be included in the ProMEX mass spectral library as unknowns. This allows recognizing these spectra again in new analyses which is not possible by using algorithms based on genome-derived theoretical mass spectra. Another potential advantage of a mass spectral reference database lies in the identification of phosphorylated peptides which usually give very ambiguous results in genome-based database searches [11,16]. For proof of concept, we included CID-MS of synthetic phosphopeptides in the ProMEX reference database which were based on *in vivo *and *in vitro *protein phosphorylation sites detected in *Arabidopsis thaliana *samples [11,17-19]. Subsequently, we were able to identify these phosphopeptides in a set of different plant samples. Several of these peptides were not identified reproducibly with genome-based search algorithms like SEQUEST.

### Protein quantification using ProMEX

ProMEX provides a quantitative estimate for the abundance of a protein/peptide in the sample. In case a complete LC/MS run is uploaded in mzData-format the ProMEX result table has an entry for each protein/peptide how often it was identified. This measure is similar to spectral count (SC) [4,15] and has been shown to be a valuable parameter for semi-quantitative mass spectrometry analysis.

### Identifying bottlenecks and limits

Setting the threshold too high or uploading LC/MS runs with large numbers of mass spectra results in computation times proportional to the number of submitted spectra in parallel to an overwhelming number of matches which is difficult to handle using a web server and HTMLformatted output. It may also appear that implementing such a tool with high computational demands as a web-based service may not be the best choice and client-site applications may be more suitable. On the other hand, this approach requires only a web browser to be accessible for the research community, thereby facilitating access to large amounts of available reference data. Some of the shortcomings could be addressed by utilizing a computer cluster and parallelising the task of scoring or by limiting the allowed file upload size. However, data file sizes will likely increase in the future. Therefore, restricting to file size may not be a viable option. Another option might be to implement a Microsoft Windows binary for a local computer utilization.

The ProMEX library will be extended by the ongoing proteomic analyses. Considering quality assurance reasons, an automated upload facility is not yet intended. However, linking ProMEX to other databases with similar background in plant proteomics research as well as databases such as peptide atlas  is an ongoing project and greatly appreciated by the authors. Further, other groups are encouraged to submit their data directly to the ProMEX database curators for inclusion. In the future we aim to apply further quality algorithms to improve the library based on spectral replicate analyses.

### Interconnecting different molecular levels through databases – a systems biology approach

As described earlier, ProMEX provides also metainformation for every mass spectrum, thereby allowing the user to trace back the experimental origin of the protein appearance. Additionally, the protein identification is linked to transcript and metabolite data via CSB.DB and the Metabolome database (GMD) [1,20]. Gene expression databases such as AtGenExpress can be directly searched with AGI codes of identified proteins looking for correlations with other genes. Metabolite data together with experimental data and reaction pathways are linked automatically via the AGI code of the identified protein. By accessing this interconnected information network data interpretation becomes more convenient. Even more important is that due to the unbiased nature of such large-scale data sets the immediate understanding is complicated. Therefore data storage in a useful way is in our experience indispensable to allow subsequent interpretation in the light of new biochemical knowledge. Thus, the data have to be made available for the community to revalidate, reconfirm or explore new information. These linked databases are probably one of the most important challenges for systems biology in the future.

## Conclusion

Here we present the plant proteomics reference database ProMEX. The database consists of tryptic peptide/phosphopeptide fragmentation mass spectra and has the potential to include all different kinds of mass spectra as demonstrated with phosphorylation site mass spectra. Search algorithms are implemented which allow the identification of proteins/peptides based on mass spectral library matching. In case complete LC/MS runs are uploaded as mzData formats it is possible to obtain semiquantitative information in form of the cumulative sum of spectra per peptide and protein. The database allows text searches based on metadata like experimental information of the samples, mass spectrometric instrument parameters or unique protein identifier like AGI codes. ProMEX integrates proteomics data with other levels of molecular organization by linking the peptide-identification to metabolite, pathway, and transcript databases. Based on these capabilities we will exploit the search function of the database in future and extend the approach to metabolite spectra.

## Availability and requirements

Project name: ProMEX

Project home page: 

Operating system(s): Available as web-based service, accessible via any web-browser

License: The service is freely available.

The BioMOBY web service *getTrypticPeptideSequenceByAGI *is listed in mobycentral under the creator *bioinformatics.mpimpgolm.mpg.de*.

The content of the peptide and experiment tables will be made available upon request either as *mdf *file for a Microsoft SQL Server Express database (freely downloadable) or as comma separated value (*csv*) file.

## Authors' contributions

JH implemented the database and the search algorithms, and wrote parts of the manuscript. SW tested the service, validated the results and wrote parts of the manuscript. MN selected the mass spectra, assisted to set up the database and wrote parts of the manuscript. DS was involved in the design of the project. JS and DW supervised the project and wrote parts of the manuscript. WW conceived and initiated a mass spectral reference library for the investigation of experimental mass fragment intensity distributions and compound identification, designed the study, and wrote parts of the manuscript.
